# Dual pH/Redox-Responsive Mixed Polymeric Micelles for Anticancer Drug Delivery and Controlled Release

**DOI:** 10.3390/pharmaceutics11040176

**Published:** 2019-04-11

**Authors:** Yongle Luo, Xujun Yin, Xi Yin, Anqi Chen, Lili Zhao, Gang Zhang, Wenbo Liao, Xiangxuan Huang, Juan Li, Can Yang Zhang

**Affiliations:** 1 School of Chemical Engineering and Energy Technology, Dongguan University of Technology, Dongguan 523808, China; luoyongle1988@gmail.com (Y.L.); yinxj96@gmail.com (X.Y.); yx3213331829@gmail.com (X.Y.); c18928489596@gmail.com (A.C.); 2017175@dgut.edu.cn (L.Z.); zhanggang@dgut.edu.cn (G.Z.); liaowenbo110@163.com (W.L.); 2 Safety Evaluation Department, Guangdong safety production technology center Co. Ltd., Guangzhou 510075, China; 3 Advanced Research Institute for Multidisciplinary Science, Beijing Institute of Technology, Beijing 100081, China; jli@bit.edu.cn

**Keywords:** mixed polymeric micelles, pH/redox-responsive, drug delivery, controlled release, anticancer

## Abstract

Stimuli-responsive polymeric micelles (PMs) have shown great potential in drug delivery and controlled release in cancer chemotherapy. Herein, inspired by the features of the tumor microenvironment, we developed dual pH/redox-responsive mixed PMs which are self-assembled from two kinds of amphiphilic diblock copolymers (poly(ethylene glycol) methyl ether-b-poly(β-amino esters) (mPEG-b-PAE) and poly(ethylene glycol) methyl ether-grafted disulfide-poly(β-amino esters) (PAE-ss-mPEG)) for anticancer drug delivery and controlled release. The co-micellization of two copolymers is evaluated by measurement of critical micelle concentration (CMC) values at different ratios of the two copolymers. The pH/redox-responsiveness of PMs is thoroughly investigated by measurement of base dissociation constant (p*K*_b_) value, particle size, and zeta-potential in different conditions. The PMs can encapsulate doxorubicin (DOX) efficiently, with high drug-loading efficacy. The DOX was released due to the swelling and disassembly of nanoparticles triggered by low pH and high glutathione (GSH) concentrations in tumor cells. The in vitro results demonstrated that drug release rate and cumulative release are obviously dependent on pH values and reducing agents. Furthermore, the cytotoxicity test showed that the mixed PMs have negligible toxicity, whereas the DOX-loaded mixed PMs exhibit high cytotoxicity for HepG2 cells. Therefore, the results demonstrate that the dual pH/redox-responsive PMs self-assembled from PAE-based diblock copolymers could be potential anticancer drug delivery carriers with pH/redox-triggered drug release, and the fabrication of stimuli-responsive mixed PMs could be an efficient strategy for preparation of intelligent drug delivery platform for disease therapy.

## 1. Introduction

With the rapid development of nanotechnology, a series of drug delivery systems (DDSs) such as liposomes [1], gels [2], polymeric micelles (PMs) [3,4], and nanoparticles (NPs) [5], etc., have been reported in cancer therapy [6]. However, major clinical barriers such as low accumulation at the tumor site, uncontrolled drug release, severe adverse effects, and high multidrug resistance still limit the efficacy of anticancer drugs and obstruct the step towards better cancer treatment [7,8,9]. To overcome these obstacles, efficient nanovehicles, which can efficiently deliver anticancer drug to tumor site with controlled drug release performance and enhanced therapeutic efficacy, urgently need to be developed. Among the aforementioned nanocarriers, PMs, which are self-assembled from amphiphilic copolymers, have shown great potential in anticancer drug-targeted delivery and controlled release due to superior advantages of technical ease, high drug-loading efficacy and biocompatibility, low cytotoxicity, and reduced side-effects [10,11,12].

The tumor metabolic profile is different from that of normal tissues, resulting in lots of features which are used as important hallmarks. For example, the pH value in the tumor microenvironment (TME) is generally lower than that in normal sites due to the elevated levels of lactic acid caused by poor oxygen perfusion [13,14]. Besides the weakly acidic conditions in the TME, the reductive characteristics of tumoral cytoplasm have attracted more and more attention in recent years [15,16,17,18]. As reported, the cellular glutathione (GSH) levels in solid tumors are much higher (~1000-times) than in normal cells [19,20]. Inspired by these specific features in the TME, a series of multi-functional stimuli-responsive PMs have been designed and prepared for drug targeted delivery and controlled release in cancer treatment [21,22,23,24,25]. For instance, Silva et al. reported a novel PM based on an amphiphilic derivative of chitosan-containing quaternary ammonium and myristoyl groups that might be a potential nanocarrier for curcumin in cancer therapy [26]. Zhang et al. designed and synthesized a novel pH-sensitive amphiphilic copolymer which could self-assemble into PMs together with a hydrophobic anticancer drug for targeted delivery and controlled release. The in vitro results demonstrated that the pH-responsive PMs may be a promising nanocarrier for encapsulated anticancer drug in cancer chemotherapy [14]. Lee’s group developed redox/pH-responsive PMs self-assembled from amphiphilic copolymer poly(β-amino ester)-grafted disulfide methylene oxide poly(ethylene glycol) (PAE-g-DSMPEG), used as anticancer drug carriers in cancer chemotherapy [19]. Johnson and co-workers synthesized a series of bioreducible and pH-responsive zwitterionic/amphiphilic block copolymers bearing a degradable disulfide linker used as dual-stimuli-responsive drug delivery vehicle for a chemotherapeutic drug [27]. In addition, various stimuli-responsive PMs which can respond to other specific cues, such as dual pH/thermal-responsiveness [28,29] and dual photo/redox-responsiveness [30] in the TME, have also been thoroughly investigated and used as drug nanocarriers in cancer chemotherapy.

**Figure 1:**
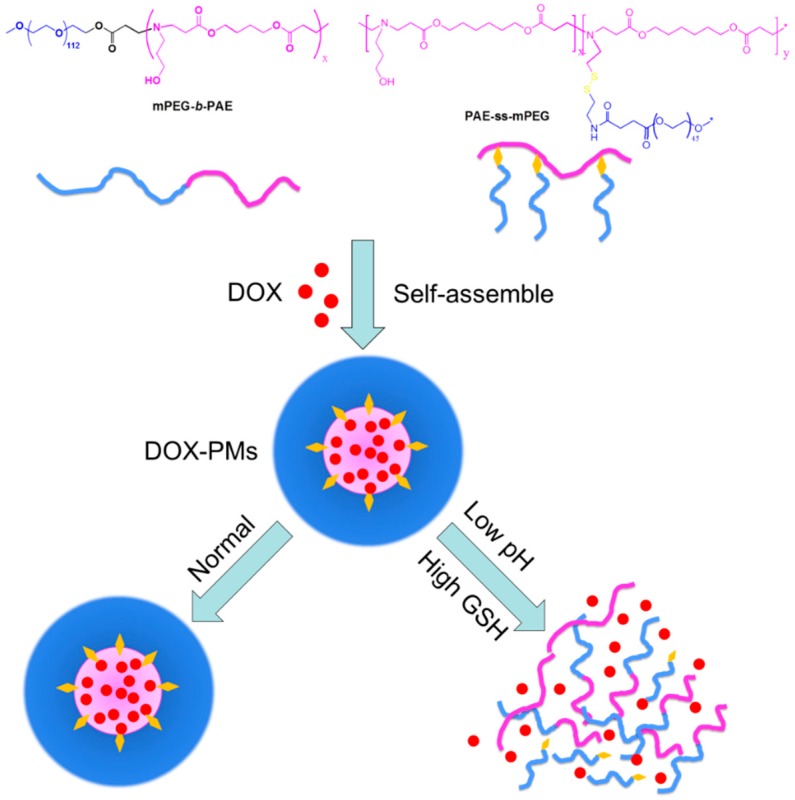
Co-micellization of pH/redox-responsive diblock copolymers for drug delivery and controlled release triggered by pH and glutathione (GSH). DOX: doxorubicin; PMs: polymeric micelles.

Herein, we design and prepare dual pH- and redox-responsive PMs which are self-assembled from two diblock copolymers: (1) pH-responsive copolymer poly(ethylene glycol) methyl ether-b-poly(β-amino esters) (mPEG-b-PAE); and (2) redox-responsive copolymer poly(ethylene glycol) methyl ether-grafted disulfide-poly(β-amino esters) (PAE-ss-mPEG). pH-sensitive segments form the polymeric micellar core, and the PEG shells are surrounded on the surface. Disulfide bonds are able to respond to reduction cues in the TME, such as GSH. Doxorubicin (DOX), which has been used extensively in various cancers as chemotherapy, is used as the model anticancer drug. As shown in Figure 1, two kinds of diblock copolymers are able to self-assemble into PMs, and DOX could be efficiently encapsulated into the core of mixed PMs (called DOX-PMs). The DOX-PMs are able to respond to the acid and GSH in the TME because of deprotonation/protonation (in acid conditions) of tertiary amine residues in the PAE segment and cleavage of disulfide bonds, respectively, resulting in rapid drug release from the PMs due to swelling and demicellization of the system. Furthermore, the other physicochemical characteristics of systems with different ratios of two kinds of diblock copolymers, including particle size, zeta-potential, loading efficacy, and cytotoxicity, are evaluated.

## 2. Materials and Methods

### 2.1. Material

Poly(ethylene glycol) methyl ether-grafted disulfide-poly(β-amino esters) (PAE3100-ss-mPEG2000) and poly(ethylene glycol) methyl ether-b-poly(β-amino esters) (mPEG5000-b-PAE4090) were synthesized as reported in our previous works [15,31]. Doxorubicin hydrochloride (DOX-HCl) was purchased from Wuhan Yuan Cheng Gong Chuang Co. Ltd. (Wuhan, China). Triethylamine (TEA, >99%), pyrene (99%), DL-dithiothreitol (DTT, which was used to replace GSH in this study), dichloromethane (DCM), dimethyl sulfoxide (DMSO), chloroform, and all other chemical reagents were used as received. Methylthiazoltetrazolium (MTT) was purchased from Sigma-Aldrich (St. Louis, MO, USA). Dulbecco’s modified eagle media (DMEM) growth media, fetal bovine serum (FBS), trypsin, penicillin, and streptomycin were all purchased from Invitrogen (Carlsbad, NM, USA); HepG2 cell lines were obtained from the American Type Culture Collection (ATCC, Manassas, MA, USA) and all other reagents were used as received.

### 2.2. Preparation of Mixed PMs and DOX-PMs

The mixed PMs self-assembled from pH-sensitive and redox-responsive diblock copolymers were prepared using a dialysis method. In a typical experiment, the diblock copolymers (mPEG-b-PAE:PAE-ss-mPEG at mass ratios of 2:1, 1:1, or 1:2, here referred to as PMs-1, PMs-2, and PMs-3, respectively) were dissolved in 40 mL of DMSO with vigorous stirring for 2 h. The resulted copolymer solution was then transferred to a dialysis bag (Molecular weight cut-off MWCO 3500–4000) and dialyzed against 1 L deionized water at pH 7.4 for 48 h at room temperature. The deionized water was replaced every 2 h for the first 12 h and then every 6 h. After filtration using 0.45-µm filter and lyophilization, the mixed PMs were obtained in powder and stored at −20 °C for further experiments.

The DOX-loaded PMs (called DOX-PMs) were prepared similarly. In brief, 40 mg of mixed two diblock copolymers at different ratios and DOX (10 mg, 20 mg, or 40 mg) were dissolved in 40 mL DMSO, and the solution was transferred to a dialysis bag. The dialysis process was carried out as aforementioned. After filtration and lyophilization as aforementioned, the DOX-PMs were obtained and stored at −20 °C for further experiments.

### 2.3. Characterization

The hydrodynamic diameter of PMs or DOX-PMs was measured by dynamic light scattering (DLS, Malvern Zetasizer Nano S, Malvern, UK). Briefly, the PMs were dissolved in phosphate buffer solution (PBS) at pH 8.0, 7.4, 6.5, 6.0, or 5.0 with or without DTT (10 mM) at a concentration of 1.0 mg/mL. As reported, a buffer solution with the addition of 10 mM DTT is commonly used to simulate the reductive microenvironment in tumor cells [32,33,34,35]. The samples were measured in a 1.0 mL quartz cuvette using a diode laser of 670 nm at room temperature. To evaluate the serum stability, the PMs were re-suspended into PBS with 20% FBS at a concentration of 1 mg/mL. After incubation at 37 °C for different time, the particle size of the sample was measured.

The morphology of PMs was determined by transmission electron microscopy (TEM, Hitachi H-7650, Hitachi-Science&Technology, Tokyo, Japan) with an acceleration voltage of 80 kV. The samples were prepared from PM solution at a concentration of 1 mg/mL onto copper grids coated with carbon. Briefly, the PM solution was re-suspended and dropped on the copper grid at atmospheric pressure and room temperature for 2 h. After drying, the sample was observed by TEM.

### 2.4. Drug Loading Efficacy

The drug loading content (LC) and entrapment efficiency (EE) were confirmed by a UV-vis spectrophotometer (UV-2450, Shimadzu, Japan) at 480 nm. In brief, 1 mg of DOX-PM powder was dissolved into 10 mL of dimethyl formamide (DMF) with vigorous stirring for 1 h. The DOX concentration of sample was measured and calculated according to the standard curve of pure DOX/DMF solution. The LC was defined as the weight ratio of encapsulated DOX to the DOX-PMs. The EE was defined as the weight ratio of encapsulated DOX to DOX in feed when preparation of DOX-PMs.

### 2.5. Critical Micelle Concentration (CMC) Measurement

The CMC values of the system (mixed diblock copolymers) were determined by the fluorescence probe technique using pyrene as a fluorescence probe. The two diblock copolymer mixtures at different ratios were first dissolved into acetone and then diluted by deionized water at a final concentration of 0.1 mg/mL. The acetone was removed using rotary evaporation with stirring for 4 h at room temperature. A series of copolymer solutions at concentrations from 0.0001 to 0.1 mg/mL were prepared. Pyrene/acetone solution (0.1 mL) was added to every vial and the acetone was allowed to evaporate to form a thin film at the bottom of the vial. The final concentration of pyrene was 6 × 10^−7^ M in water. The mixed solution was equilibrated at room temperature for 24 h in dark. And then, the fluorescence spectra of samples were obtained using a fluorescence spectrophotometer (F-4500, Hitachi-Science&Technology, Hitachi, Japan) with an emission wavelength of 373 nm.

### 2.6. Potentiometric Titration

To measure the base dissociation constant (p*K*_b_) of system, potentiometric titrations were operated as reported. In brief, the mixed diblock copolymers were dissolved in deionized water, and the pH was adjusted to 3.0 with dilute hydrochloric acid. Then, NaOH solution (0.1 mol/L) was added dropwise in the mixed solution, and the real-time pH values were recorded by an automatic titration titrator (Hanon T-860, Jinan Hanon Instruments Co., Ltd., Jinan, China). The p*K*_b_ value of system was determined according to the plots of pH value against the volume of NaOH solution.

### 2.7. pH and Redox Responsiveness

To evaluate the pH- and redox-responsiveness of system, the PMs were firstly re-suspended in PBS at different pH values with or without DTT (10 mM). After incubation for 4 h at 37 °C, the hydrodynamic diameter of sample was measured by DLS as aforementioned.

### 2.8. In Vitro Release of DOX from PMs

The in vitro release of DOX from DOX-PMs was recorded using UV-vis spectrophotometer. To acquire sink conditions, in vitro drug release test was performed at low drug concentrations. In brief, 5 mg DOX-PMs were dissolved into 5 mL in PBS at pH 7.4 or 6.0 with or without the addition of DTT (10 mM), and the solution was transferred into a cellulose dialysis bag (MWCO 3500–4000). Then, the dialysis bag was placed in corresponding buffer (45 mL) in a beaker. The experiment was carried out at 37 °C with stirring at 110 rpm. At the desired time, 1 mL of solution was taken for measurement using UV-vis spectrophotometry, and 1 mL of fresh PBS was added. The cumulative drug release percent (*E*_r_) was calculated according to our previous work [14]. Equation (1) is shown as follows:

(1) $$E_{r}(\%)=\frac{V_{e}\sum_{1}^{n-1}C_{i}+V_{0}C_{n}}{m_{DOX}}\times 100\%$$

where *m*_DOX_ is the amount of encapsulated drug in PMs, *V*_e_ is the volume of buffer in the dialysis bag, *V*_0_ is the total volume of buffer in the beaker (50 mL), and *C*_i_ is the DOX concentration in the *i*th sample.

### 2.9. Cell Culture

The HepG2 cells were cultured in DMEM supplemented with 10% FBS, 100 units/mL penicillin, and 100 µg/mL streptomycin. The cells were incubated at 37 °C in a CO_2_ (5%) incubator.

### 2.10. Cytotoxicity Test

The cytotoxicity of free DOX, blank PMs, and various DOX-PMs against HepG2 cells were evaluated by standard MTT assay [36,37,38,39]. In brief, HepG2 cells were seeded into a 96-well plate at an initial density of 1 × 104 cells/well in 200 μL DMEM medium and cultured in incubator for 24 h. The medium was removed, and 200 µL/well of free DOX, blank PMs, and DOX-PMs with different concentrations of DOX were added and cultured for 24 h. The wells without cells were used as blank, and the wells with cells but without treatment were used as control. After addition of 20 µL of MTT solution, the plate was shaken for 5 min at 150 rpm and then cultured for 4 h in incubator. After discarding the culture supernatants, 200 µL of DMSO were added to each well. The plate was gently agitated for 15 min, and the absorbance of sample was recorded by a microplate reader (Multiskan Spectrum, Thermo Scientific, Vantaa, Finland) at 490 nm. The cell viability (%) was defined as the absorbance ratio of difference between sample and blank and difference between control and blank.

### 2.11. Statistical Analysis

The experimental data were presented with an average values, expressed as the mean ± standard deviation (S.D.). Statistical analysis was conducted using two-sample Student’s *t*-test of origin 8.5, and considered to be significant when *p* < 0.05.

## 3. Results and Discussions

### 3.1. Preparation and Chacracterization of PMs and DOX-Loaded PMs

**Figure 2:**
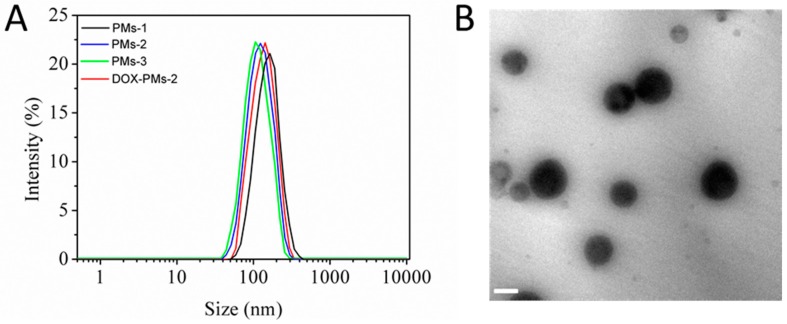
(**A**) Hydrodynamic diameter of different mixed PM and DOX-loaded PMs-2 measured by dynamic light scattering (DLS). (**B**) TEM image of DOX-PMs-2 after incubation in PBS at pH 7.4 for 2 h. Scale bar, 100 nm.

Blank mixed PMs and DOX-loaded mixed PMs were prepared by the dialysis method. The particle size and morphology were measured and characterized by DLS (Figure 2A) and TEM (Figure 2B), respectively. As shown in Figure 2A, the particle sizes of mixed PMs-1, PMs-2, and PMs-3 were 160.7 nm, 138.6 nm, and 115.1 nm, respectively. The reason could be the much larger polymeric micellar core with increasing PAE segment in the system when the ratios of the linear diblock copolymer mPEG-b-PAE were enhanced. The particle size of DOX-PMs-2 (mixed copolymers:DOX = 2:1, mass ratio) was slightly higher (148.0 nm) than that of PMs-2 due to the loading of hydrophobic DOX molecules in the micellar core. In addition, the stability of three types of PMs in PBS containing 20% FBS at pH 7.4 was evaluated via the change of particle size, as shown in Figure S1 (Supporting Information). The results demonstrated that all of three mixed PMs showed high serum stability after incubation for 5 days. That indicated three mixed PMs possessed the potential to prolong the circulation time, thereby improving the accumulation of PMs in the site of tumor by enhanced permeability and retention (EPR) effect. Figure 2B presents the TEM images of DOX-PMs-2 after incubation in PBS at pH 7.4 for 2 h. The particle size was approximately 143.4 nm, and DOX-PMs-2 exhibited a uniformly spherical in shape with good dispersibility. The particle size measured by TEM was slightly lower compared with that determined by DLS, resulting from the shrinking of the polymeric micelles during drying process prior to TEM imaging. The TEM images of DOX-PMs-1 and DOX-PMs-3 are shown in Figure S2 (Supporting Information), and similar results were observed. 

The particle size, polydispersity index (PDI), LC, and EE of the three types of DOX-PMs at different mass ratios of drug and carriers are shown in Table 1. As expected, the particle sizes of DOX-loaded PMs were increased compared with those of blank PMs. With increasing DOX in feed, the particle size was also enhanced due to more DOX molecules being encapsulated in the micellar core. When the mass ratio of drug and carriers was increased from 1:4 to 1:1, the LC was enhanced sharply and then tended to be gentle, while the EE was enhanced firstly and then reduced rapidly caused by the limitation of drug-loading capability of mixed PMs. Besides, at the same mass ratio of drug and carriers, the mixed PMs-1 had the highest drug loading efficacy, attributed to the much bigger micellar core. Therefore, DOX-PMs at the drug:carrier mass ratio of 1:2 for the three types of mixed PMs were selected for further study.

### 3.2. CMC Measurement

**Figure 3:**
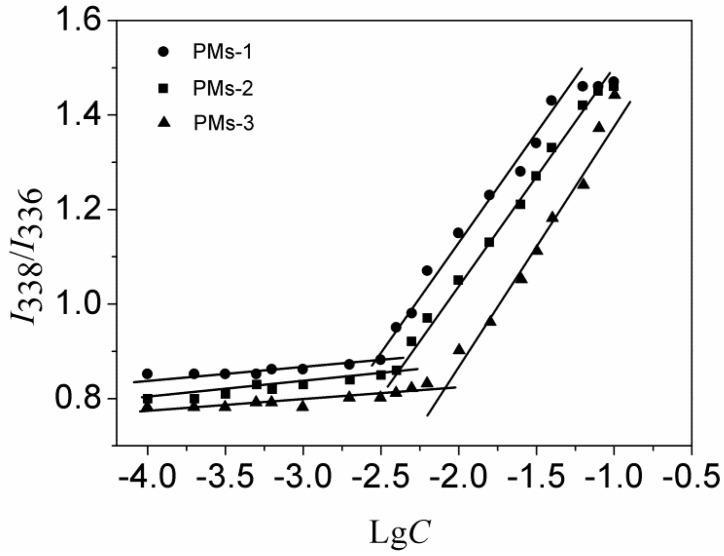
Plot of intensity ratios (*I*_338_/*I*_336_) as a function of logarithm of the mixed copolymers at various concentrations (mg/mL).

The CMC value is related to the thermodynamic stability of polymeric micelles and affects the initial release of the drug when introduced into the bloodstream by intravenous administration. The low CMC value indicated the system could self-assemble easily into polymeric micelles. The CMC values of three types of mixed systems were measured by fluorescence spectroscopy using pyrene as the probe, as shown in Figure 3. The CMC values of mixed PMs-1, PMs-2, and PMs-3 were determined as 3.1 mg/L, 4.2 mg/L, and 6.4 mg/L, respectively, which were values much lower than those of PMs self-assembled from single amphiphilic copolymer [40], indicating the much higher stability. Furthermore, the result showed that the stability of mixed PMs-1 is slightly superior to PMs-2 and PMs-3. The reason could be that a lower CMC value was resulted from the more hydrophobic PAE segment in mixed diblock copolymer. In summary, the three types of mixed copolymers were able to self-assemble into mixed polymeric micelles with low CMC values, indicating that these PMs could be potential efficient hydrophobic drug carriers with high stability.

### 3.3. pH Sensitivity of Three Types of PMs

**Figure 4:**
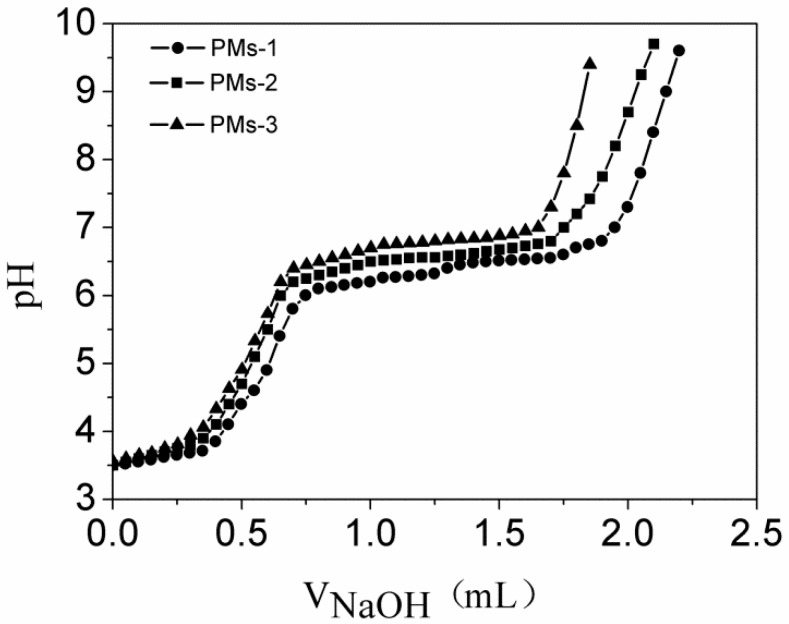
The potentiometric titration of the mixed copolymer solution with the mass ratios of mPEG-b-PAE and PAE-ss-mPEG at 2:1, 1:1, and 1:2. PAE-ss-mPEG: poly(ethylene glycol) methyl ether-grafted disulfide-poly(β-amino esters); mPEG-b-PAE: poly(ethylene glycol) methyl ether-b-poly(β-amino esters).

The p*K*_b_ value of mixed PMs was defined as the pH value at 50% neutralization of protonated amine groups according to the reference [41]. Here, the p*K*_b_ values of the three types of system were measured by acid–base titration, and the corresponding titration curves are shown in Figure 4. As expected, the pH value increased sharply with the addition of NaOH solution, then reached a plateau, and then increased rapidly again. The reason could be that the tertiary amine residues in the PAE segment were protonated in acidic environment and were transferred to deprotonation in basic environment. As shown in Figure 4, the p*K*_b_ values of PMs-1, PMs-2, and PMs-3 were measured as 6.45, 6.57, and 6.72, respectively, owing to different amount of pH-sensitive PAE segment in the system. PMs-1 showed the lowest p*K*_b_ value due to the ratio of diblock copolymer mPEG-b-PAE in the mixed system. With the increase of diblock copolymer PAE-ss-mPEG, the p*K*_b_ value of sysem increased from 6.45 to 6.72. The results suggested that p*K*_b_ values of three types of mixed PMs were in the range of weakly acidic range, indicating the suitable and potential pH-responsiveness of mixed PMs used as anticancer drug carriers.

### 3.4. pH- and Redox-Responsiveness

**Figure 5:**
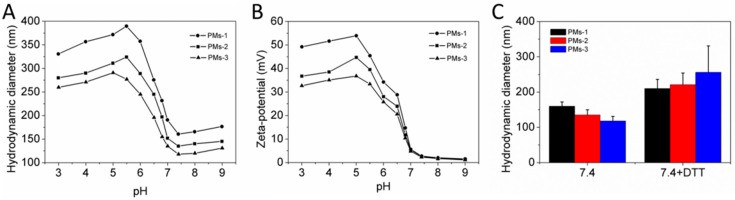
Particle size (**A**) and zeta-potential (**B**) of the mixed PM dependence on pH value in PBS. (**C**) Particle size of the mixed PMs in PBS with or without DTT (10 mM) after incubation for 2 h.

Next, the pH- and redox-responsiveness of mixed PMs were evaluated through measurement of size and zeta-potential change of system at different pH conditions, as shown in Figure 5. Figure 5A shows the particle size of three mixed PMs depended on the pH value. When the pH of the mixed diblock copolymer solution was higher than 7.0, the particle sizes of the three mixed PMs increased slightly with the pH increase. The reason could be the few tertiary amine residues in the PAE segment with protonation, resulting in slight swelling of PMs. When the pH value decreased to the range of 7.0–5.5, the particle sizes of three mixed PMs increased sharply. The reason may be that the tertiary amine residues in PAE segment were fully protonated in acidic conditions, leading to the transition from hydrophobic PAE to a hydrophilic one that transformed the PMs from dense to swollen structures, so that the particle size was increased. The PMs-1 with the most tertiary amine residues were the biggest and exhibited the most dramatic size change compared with the other two mixed PMs. As expected, the PMs-3 with the lowest segment ratio of PAE had the smallest particle size and change, consistent with the results in Figure 2. Thus, the more pH-sensitive and hydrophobic PAE content, the greater the micelle particle size and the greater the size change when pH decreased from base to acid. The reason could be that the tertiary amine residues in the PAE segment were transferred from deprotonated to protonated, resulting in a hydrophilic PAE segment in the system and swollen nanoparticles. When the pH value decreased below pH 6.0 sequentially, the particle sizes of three types of mixed PMs were reduced slightly because of disassembly of few polymeric micelles. Figure 5B shows the zeta-potential of PMs at different pH conditions. The zeta-potential of three mixed PMs increased significantly with pH value decrease as a result of the tertiary amine residues in the PAE segment being transferred from deprotonation to protonation. The zeta-potential was positive, indicating the high cellular uptake due to the charge interactions, as reported in references [42,43]. When the pH was higher than 7.0, the zeta-potential was decreased with the pH increase due to the uncharged PEG shield on the surface of the polymeric micelles. In summary, three types of mixed PMs showed effective pH sensitivity. The redox-responsiveness of PMs was next investigated, as shown in Figure 5C. After incubation in PBS with DTT (10 mM) for 2 h, particle sizes of three types of mixed PMs were obviously increased, attributed to the cleavage of disulfide bonds which resulted in detachment of hydrophilic PEG segment that might lead to the aggregation of nanoparticles. Furthermore, the left hydrophobic PAE segment was entrapped into the micellar core, which led to the increase of particle size. PMs-3 with the most diblock copolymer brush PAE-ss-mPEG, including disulfide bonds, showed much greater size changes compared to the other mixed PMs. In conclusion, the prepared three mixed PMs showed pH- and redox-responsiveness.

### 3.5. pH- and Redox-Triggered DOX Rlease In Vitro

**Figure 6:**
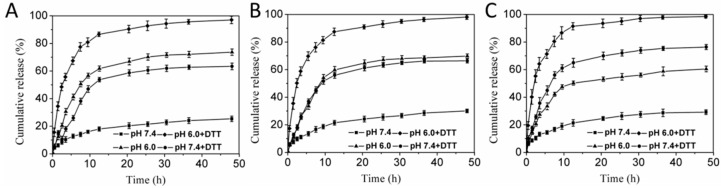
In vitro drug release profiles of DOX-loaded PMs-1 (**A**), DOX-loaded PMs-2 (**B**), or DOX-loaded PMs-3 (**C**) in PBS at pH 7.4, pH 6.0, pH 7.4 with 10 mM DTT, and pH 6.0 with 10 mM DTT (*n* = 3, mean ± SD).

After effective accumulation of drug-loaded system at the targeted site, the controlled drug release from carries triggered by specific microenvironmental cues are of great importance. Next, the in vitro DOX release from mixed PMs in different conditions (pH 7.4, pH 6.0, pH 7.4 with DTT and pH 6.0 with DTT) was investigated, as shown in Figure 6. It could be observed that the release rates of DOX from PMs were markedly influenced by pH values and DTT. At pH 7.4, the mixed PMs were tight and compact; the release rates of DOX were very slow for the three DOX-PMs. The cumulative release of DOX was less than 30% after 48 h for DOX-PMs-1, DOX-PMs-2, and DOX-PMs-3, indicating that the DOX molecules could be well protected in the micellar core and with reduced burst release. When the pH decreased to 6.0, the DOX release rate was obviously accelerated, and the cumulative release of DOX was approximately 70%, 67%, and 60% after 48 h for DOX-PMs-1 (Figure 6A), DOX-PMs-2 (Figure 6B), and DOX-PMs-3 (Figure 6C), respectively, due to the swelling of polymeric micelles caused by deprotonation/protonation (in acid conditions) of tertiary amine residues in PAE segment. The cumulative release of DOX for DOX-PMs-3 was the highest, attributed to the greater PAE segment in the system compared to the others. At pH 7.4 with DTT, the drug release rates and cumulative release were also significantly improved, resulting from the cleavage of disulfide bonds and the detachment of the PEG segment which led to the increase in porosity. Moreover, the cumulative release of DOX at 48 h for DOX-PMs-3 (75%, Figure 6C) was higher compared to DOX-PMs-1 (63%, Figure 6A) and DOX-PMs-2 (65%, Figure 6B), due to the higher mass ratio of diblock copolymer PAE-ss-mPEG in the system. At pH 6.0 with DTT, the DOX release rates of three DOX-loaded PMs were obviously enhanced, and the cumulative release of DOX was almost 100% for three DOX-PMs, caused by the acid and DTT in the solution. In summary, the DOX was of controlled release from the mixed PMs triggered by the pH and DTT, indicating the DOX might have controlled release in the tumor microenvironment by responding to the acid and reducing agent glutathione (GSH).

### 3.6. Cytotoxicity Assay

**Figure 7:**
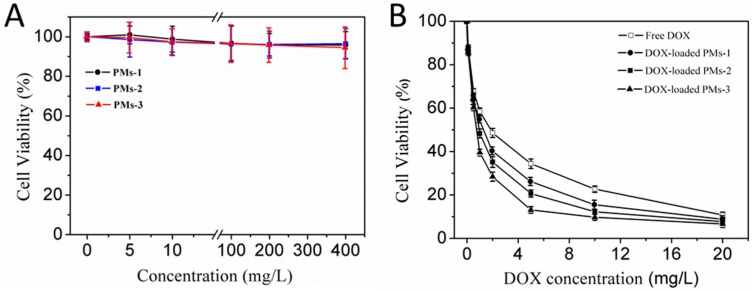
In vitro cytotoxicity of blank three PMs (**A**) and DOX-loaded PMs (**B**) at different concentrations in HepG2 cells after incubation for 24 h.

Next, the cytotoxic effects of the blank PMs, free DOX, and DOX-PMs for HepG2 cells were evaluated using MTT assay, as shown in Figure 7. Since their cytotoxic effect increased slightly with the increasing PM concentration after incubation of 24 h, the cell viability for treatment of PMs-1, PMs-2, and PMs-3 was higher than 95% even at the highest concentration of PMs (400 mg/L) (Figure 7A). The result demonstrated that all of three types of mixed PMs had negligible cytotoxicity for HepG2 cells. Figure 7B shows the cytotoxicity of free DOX and three DOX-PMs against HepG2 cells for 24 h. The half maximal inhibitory concentration (IC50) values of free DOX, DOX-PMs-1, DOX-PMs-2, and DOX-PMs-3 were measured as 1.85 mg/L, 1.50 mg/L, 0.91 mg/L, and 0.75 mg/L, respectively. The cytotoxicity of DOX-PMs for HepG2 cells was higher than that of free DOX, possibly resulting from the enhanced cellular uptake and reduced active efflux of DOX molecules. Compared with the other DOX-PMs, the DOX-PMs-3 showed the highest cytotoxicity against HepG2 cells due to the rapid drug release rate and high cumulative release at 24 h, as shown in Figure 6. Conclusively, the three mixed PMs had very low cytotoxicity and the DOX-PMs could efficiently inhibit the suppressed HepG2 cell growth.

## 4. Conclusions

Three types of PMs were self-assembled from mixture of two kinds of diblock copolymers. The particle sizes of three PMs were in the range of 100–200 nm with a spherical shape. The three types of PMs showed low CMC values, indicating the self-assembly and high stability of system in aqueous solution. DOX, one of the most effective drugs against a wide range of cancers, was efficiently encapsulated into the micellar core via the hydrophobic interaction. The pH- and redox-responsiveness of mixed PMs were thoroughly investigated by recording the particle size and zeta-potential at different conditions. In vitro drug release profiles and cytotoxicity assay demonstrated that the DOX was released from mixed PMs triggered by acidic pH and high concentration of DTT, and the released DOX molecules were able to inhibit the HepG2 cell growth. Furthermore, the structure–activity relationship of mixed PMs based on different mass ratios of two diblock copolymers were preliminarily studied. These results suggested that the dual pH- and redox-responsive polymeric micelles might be promising as a potential efficient drug delivery carrier for cancer chemotherapy, and mixed polymeric micelles self-assembled from two or more kinds of stimuli-responsive copolymers could be an effective method to prepare multi-functional drug delivery vehicles.

## Figures and Tables

**Table 1:** Particle size, polydispersity index (PDI), loading content (LC), and entrapment (EE) of DOX-PMs at different mass ratios of drug and carriers.

PMs (40 mg)	DOX (mg)	Size (nm) ^a^	PDI ^a^	LC (%) ^b^	EE (%) ^b^
PMs-1	10	165	0.25	13.60	61.18
	20	171	0.22	27.71	73.45
	40	178	0.35	28.67	53.76
PMs-2	10	143	0.21	14.21	60.43
	20	148	0.23	26.85	77.64
	40	155	0.33	29.11	55.70
PMs-3	10	121	0.23	12.77	59.08
	20	125	0.31	23.90	71.54
	40	130	0.33	25.69	52.77

^a^ measured by DLS, ^b^ measured by UV-vis.

## Supplementary Materials

The following are available online at https://www.mdpi.com/1999-4923/11/4/176/s1. Figure S1: Serum stability of three mixed PMs in the presence of 20% FBS in PBS at room temperature (*n* = 3, mean ± SD). Figure S2: TEM image of DOX-PMs-1 (left) and DOX-PMs-3 (right) after incubation in PBS at pH 7.4 for 2 h. Mixed copolymers: DOX = 2:1, mass ratio. Scale bar, 100 nm.
