# Supplementary material for: Dual pH/Redox-Responsive Mixed Polymeric Micelles for Anticancer Drug Delivery and Controlled Release
Source: Pharmaceutics. 2019 Apr 11;11(4):176. doi: 10.3390/pharmaceutics11040176 (PMC6523239; doi:10.3390/pharmaceutics11040176)
Supplement: Supplementary file 1 [file pharmaceutics-11-00176-s001.pdf]

pharmaceutics

Supplementary Materials: Dual pH/Redox-Responsive Mixed Polymeric Micelles for Anticancer Drug Delivery and Controlled Release

Yongle Luo, Xujun Yin, Xi Yin, Anqi Chen, Lili Zhao, Gang Zhang, Wenbo Liao, Xiangxuan Huang, Juan Li, and Can Yang Zhang

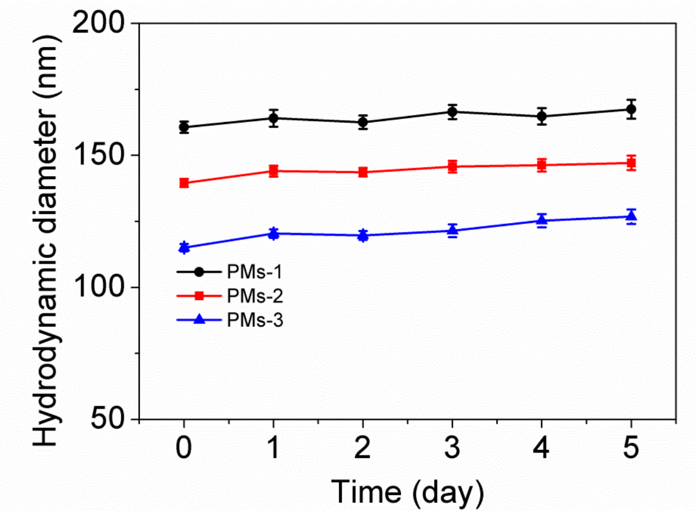

Figure S1. Serum stability of three mixed PMs in the presence of 20 % FBS in PBS at room temperature (n = 3, mean ± SD).

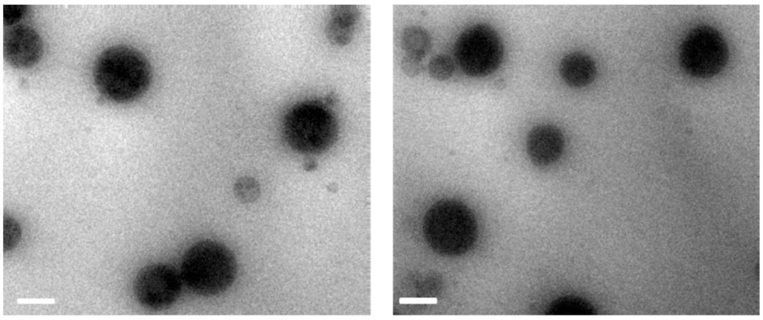

Figure S2. TEM image of DOX-PMs-1 (left) and DOX-PMs-3 (right) after incubation in PBS at pH 7.4 for 2 h. Mixed copolymers:DOX = 2:1, mass ratio. Scale bar, 100 nm.
